# Cellular mechanisms of mutations in Kv7.1: auditory functions in Jervell and Lange-Nielsen syndrome vs. Romano–Ward syndrome

**DOI:** 10.3389/fncel.2015.00032

**Published:** 2015-02-06

**Authors:** Atefeh Mousavi Nik, Somayeh Gharaie, Hyo Jeong Kim

**Affiliations:** Department of Anesthesiology and Pain Medicine, Center for Neuroscience, School of Medicine, University of California, DavisDavis, CA, USA

**Keywords:** genetic diseases, membrane trafficking, mutant, potassium channels, hearing loss

## Abstract

As a result of cell-specific functions of voltage-activated K^+^ channels, such as Kv7.1, mutations in this channel produce profound cardiac and auditory defects. At the same time, the massive diversity of K^+^ channels allows for compensatory substitution of mutant channels by other functional channels of their type to minimize defective phenotypes. Kv7.1 represents a clear example of such functional dichotomy. While several point mutations in the channel result in a cardio-auditory syndrome called Jervell and Lange-Nielsen syndrome (JLNS), about 100-fold mutations result in long QT syndrome (LQTS) denoted as Romano–Ward syndrome (RWS), which has an intact auditory phenotype. To determine whether the cellular mechanisms for the diverse phenotypic outcome of Kv7.1 mutations, are dependent on the tissue-specific function of the channel and/or specialized functions of the channel, we made series of point mutations in hKv7.1 ascribed to JLNS and RWS. For JLNS mutations, all except W248F yielded non-functional channels when expressed alone. Although W248F at the end of the S4 domain yielded a functional current, it underwent marked inactivation at positive voltages, rendering the channel non-functional. We demonstrate that by definition, none of the JLNS mutants operated in a dominant negative (DN) fashion. Instead, the JLNS mutants have impaired membrane trafficking, trapped in the endoplasmic reticulum (ER) and Cis-Golgi. The RWS mutants exhibited varied functional phenotypes. However, they can be summed up as exhibiting DN effects. Phenotypic differences between JLNS and RWS may stem from tissue-specific functional requirements of cardiac vs. inner ear non-sensory cells.

## Introduction

Potassium (K^+^) channels perform diverse functions in cells, ranging from regulation of the membrane potential of excitable cells, controlling cell volume, cell division, migration and apoptotic cell death (DeCoursey et al., 1984; Bortner et al., 1997; Wei et al., 2004), as well as K^+^ outflow in specialized compartments in the body, e.g., the kidney and inner ear (Giebisch, 2001; Nin et al., 2008, 2012). Hence, after the human genome was delineated, it was reassuring to identify K^+^ channel genes as the most varied ion channel in the body (Gutman et al., 2005; Wulff et al., 2009). The cell-specific functions of K^+^ channels are such that mutations in these channels often result in a plethora of pathological conditions. This is because in K^+^ channelopathy, other K^+^ channels can hardly compensate for their loss or gain of functions. Additionally, because K^+^ channels operate in multi-component systems, it is extremely difficult to identify their subunit-specific functions. Thus, in order to define their subtype-specific functions, studies in heterologous expression systems are used routinely (Lai and Jan, 2006; Xu et al., 2007; Maffie and Rudy, 2008; Kim et al., 2011). Moreover, the ensuing properties of either the sequestered wild-type (WT) or mutant (MT) K^+^ channel would be incomplete, unless they are interpreted in the context of their cell-specific functions and other potential ion channel subunits they interact with, often promiscuously (Xu et al., 2007).

The voltage-activated K^+^ channel Kv7.1, previously called KvLQT1, is expressed in several tissues, including the heart, kidney and cells in the inner ear lateral wall. The Kv7.1 gene comprises of 404 kb at 11p15.5 (Wang et al., 1996) and codes for a 75-kDa protein, containing 676 amino acids (Yang et al., 1997). The significance of Kv7.1 functions in the heart and inner ear underscore several mutations which result in a cardio-auditory syndrome called Jervell and Lange-Nielsen syndrome (JLNS) (Jervell and Lange-Nielsen, 1957), as well as over 200 mutations that are reported to cause long QT syndrome (LQTS) without the hearing phenotype, as seen in Romano–Ward syndrome (RWS) (Morita et al., 2008; Hedley et al., 2009). Moreover, the cellular mechanisms responsible for the seemingly similar mutations in LQTS that spare inner ear diseased phenotype remain distressingly unknown.

JLNS and RWS can be found worldwide, affecting families of diverse backgrounds ranging from Asian, European and American origins. To determine the biophysical and cellular mechanisms of JLNS and RWS, and to provide mechanistic insight on the functional outputs of JLNS vs. RWS mutations, we generated several mutant forms of the human Kv7.1 (hKv7.1, *KCNQ1*) clone, using site-directed mutagenesis to define their sub-cellular localization and examined their electrophysiological properties. A caveat to consider at the onset of this study is that although Kv7.1 invariably operates together with auxiliary subunits e.g., KCNE, and several other interacting proteins, we focused mainly on the biophysical aspects of the pore-forming subunit of the channel.

We identified JLNS and RWS mutations in *KCNQ1* at the S4-S5-linker, the pore loop (P-loop) and the C-terminus of hKv7.1 which have been found to control channel gating, permeation and modulation, respectively (Lipkind et al., 1995; Schmitt et al., 2000; Choveau et al., 2011; Labro et al., 2011) (Table 1). We report that all P-loop and C-terminal mutations (seven mutations) ascribed to JLNS yielded non-functional channels when expressed alone. Moreover, the W248F at the end of the S4 domain yielded a functional current, but at positive step potentials, the current underwent marked, inactivation, rendering the channel essentially non-functional. Not only did the seven JLNS mutant channels produce non-functional channels, they also affected channel trafficking and cell-surface expression. Previous studies have indicated that some mutants such as Y461X Kimoto et al. (2013), R594Q, A178T, and A525T Harmer et al. (2014) and 1149insT Wang et al. (2011) reduce cell-surface expression due to trafficking defects.

**Table 1:** **hKv7.1 mutations in JLNS and RWS**.

**Family**	**Amino acid change**	**DNA change**	**Protein domain**	**Exon**	**References**
**JLNS**					
Japanese	W248F	GG733TC	S4	5	Franqueza et al., 1999
Finnish	T311I	C932T	Pore region	7	Saarinen et al., 1998
Chinese	T322M	C965T	Pore region	7	Napolitano et al., 2005
British	A336fs+16X	1008delC	S6- C-terminal cytoplasmic	7	Tyson et al., 2000
Swedish	R518X	C1552T	C-terminal cytoplasmic	12	Wei et al., 2000
Norway	Q530X	C1588T	C-terminal cytoplasmic	12	Tranebjaerg et al., 1999
Kabylia	E543fs+107X	1630 del7, ins8	C-terminal cytoplasmic	13	Neyroud et al., 1997
Finnish	G589D	G1766A	C-terminal cytoplasmic	15	Piippo et al., 2001
**RWS**					
Japanese	D242N	G724A	S4	5	Itoh et al., 1998
French	R243P	G728C	S4	5	Millat et al., 2006
Japanese	L250H	T749A	S4–S5 linker	5	Itoh et al., 1998
Chinese	G306V	G917T	Pore region	6	Liu et al., 2002
American	D317N	G949A	Pore region	7	Wollnik et al., 1997
American	L374fs+43X	1124del4	C-terminal cytoplasmic	8	Tester et al., 2005
Italy	N586D	A1756G	C-terminal cytoplasmic	15	Napolitano et al., 2005
American	L619M	T1855A	C-terminal cytoplasmic	16	Tester et al., 2005

On the other hand, the RWS mutants showed functional phenotypes consisting of channels with no measurable current when expressed alone, but stunted-to-no-measurable current upon addition of the WT subunit. The RWS mutants, however, produced DN effect. Previous studies reported that mutations in *KCNQ1* such as R555H Aromolaran et al. (2014), R539W Chouabe et al. (2000), and K557E Spatjens et al. (2014), cause dominant-negative effects that reduce the current density significantly. Our findings provide integrated cellular and molecular mechanisms of hKv7.1 functions and the ensuing diseased phenotype in JLNS and RWS may stem from the tissue-specific function of the channel.

## Experimental procedures

### Generation of mutant forms of hKv7.1 and epitope-tagged construct

Wild-type (WT) hKv7.1 clone (Genebank: AF000571) was kindly given to the laboratory by Dr. N. Chiamvimonvat (Sharma et al., 2004). The CDS were subcloned into a pIRES2-EGFP plasmid vector (Clonetech, Mountain View, CA). Each of the JLNS and RWS mutations were generated from WT gene using QuickChangeII mutagenesis kit (Stratagene, La Jolla, CA) and verified by automated sequencing. pIRES2-EGFP-hKv7.1-WT and mutants (MT) were used in electrophysiology/patch-clamp study using EGFP as the reporter gene. To study the subcellular localization of WT and MT subunits, two different epitopes, modified HA- and c-Myc-tags, were inserted into pCMV-hKv7.1-WT and MT constructs. Here, the EGFP genes were eliminated. Modified HA- and c-Myc epitopes were flanked with ClC-5 chloride channel D1-D2 loop to increase accessibility and inserted in the end of the S1-S2 loop of hKv7.1 as previously described in Kv7.2/7.3 and Kv7.4 channels (Schwake et al., 2000; Kim et al., 2011). S1-S2 loop amino acid sequences changed to STIEQNSEH**YP YDVPDYA**VTFEERDKCPEWN for HA-tagged constructs and STIEQNSEH**EQKLISEEDL**VTF EERDKCPEWN for c-Myc tagged constructs; whole inserted regions are underlined and epitopes are shown in bold. Epitope-tagged clones were generated by recombination polymerase chain reaction and the sequences were verified.

### Cell culture and hKv7.1 gene delivery

Chinese Hamster Ovary (CHO) cell line was used in this study. CHO cells were maintained in F-12 media with 10% fetal bovine serum (FBS) and 1x antibiotic-antimycotic mixture (Invitrogen, Carlsbad, CA) at 37°C with 5% CO_2_. CHO cells were seeded onto 12 mm coverslips in F-12 + 10% FBS without antibiotics and cultured 12–24 h before transfection. hKv7.1-WT or hKv7.1-MT DNA were transfected into cells alone or in combinations, using 200 ng/well, employing Lipofectamine 2000 (Invitrogen) procedure according to the manufacture's instruction. For experiments in which the WT and MT channels were expressed jointly, we estimated the ratio of expression based on the amount transfected relative to the total DNA (limitations of this strategy is addressed in the Discussion).

### Electrophysiological recordings

We performed these experiments using an Axopatch 200B amplifier (Axon Instruments, Inc., Union City, CA). Fire-polished electrodes (3–4 MΩ) were pulled from borosilicate glass. We recorded K^+^ currents using the whole-cell voltage-clamp configuration and recordings were performed from single, uncoupled cells at room temperature (20–22°C). The composition of electrode solution was (in mM): KCl 140, MgCl_2_ 1, HEPES 10, EGTA 10, CaCl_2_ 1, K_2_ATP 4, pH 7.2 with KOH. The bath solution contained (in mM): NaCl 145, KCl 4, CaCl_2_ 1.8, MgCl_2_ 0.5, HEPES 10, D-Glucose 5, pH 7.4 with NaOH. Unless otherwise indicated, reagents were obtained from Sigma-Aldrich (St. Louis, MO).

Outward hKv7.1 channel current traces were generated with depolarizing voltage steps from a holding voltage of −80 mV and stepped to varying step potentials (Δ V = 5–10 mV). Currents were measured after capacitance compensation and series resistance compensation (6–8 MΩ) (nominally 70–90%), and filtered at 2 kHz using an 8-pole Bessel filter, and sampled at 5 kHz. Liquid-junction potentials were less than 2 mV (1.7 ± 0.2 mV, *n* = 28). Whole-cell K^+^ current amplitude, at varying test potentials, was measured at the peak and steady-state levels using peak and steady-state detection routine. To determine the current density, the current was divided by the cell capacitance (pF) and current density was plotted against voltage. Analyses of data were performed using custom-written software and Microcal Origin (Northampton, MA) programs. Pooled data are presented as means ±SD. Multiple comparisons vs. control data were performed using *t*-test or Kruskal-Wallis one-way analysis of variance (Dunn's method). Whole-cell K^+^ tail currents were normalized and plotted against the step potential. Using the Boltzmann function I/I_max_ = 1 + [exp (V_1/2_-V)/k_m_] where V_1/2_is the half-activation voltage, k_m_ = RT/zF is the slope factor, I is the magnitude of the current, and I_max_ denotes the maximum current magnitude, we plotted and fitted the steady-state activation curves of the currents.

The α-subunit of the K^+^ channel consists of independent monomers that assemble to form a conducting pore. The stoichiometric combination of the WT and MT monomers is expected to be a random process. Thus, the probability of forming a functional channel with only WT monomers (P_WT only_) can be described by a polynomial distribution with the proportion of WT monomers (p_WT_) available to the n^th^ power (P_WT only_ = p^n^_WT_), where n is the number of monomers present in a functional channel (MacKinnon, 1991; Kim et al., 2011).

### Immunostaining

The primary antibodies used were: Rabbit polyclonal anti-hKv7.1 (Abcam, Cambridge, MA), mouse monoclonal anti-HA (Covance, Emeryville, CA), rabbit polyclonal anti-HA, chicken polyclonal anti c-Myc, rabbit polyclonal anti-Cryptochrome P450 (Abcam, Cambridge, MA), mouse monoclonal anti-early endosome antigen 1 (EEA1), mouse monoclonal anti-Golgi A4 (P230) and mouse monoclonal anti- Golgi A2 (Golgi matrix protein of 130 kD; BD Biosciences, San Jose, CA) at a final concentration of 1 μg/ml. The secondary antibodies were: donkey anti-mouse-Cy3, donkey anti-mouse-Cy5, donkey anti-rabbit-Cy3, donkey anti-rabbit-Cy5, and donkey anti-chicken-Cy5 (Jackson ImmunoResearch Laboratories Inc., West Grove, PA) in manufacturer's recommended concentrations.

CHO cells were incubated 24 h after transfection and fixed in 4% paraformaldehyde in 1X phosphate buffered saline (PBS) for 10 min, followed by series washing with PBS. To use anti-hKv7.1 antibody and to study intracellular localization, cells were permeabilized and blocked in 0.1% Triton X-100, 3% bovine serum albumin (BSA), and 5% normal donkey serum in PBS for 30 min at 37°C. To label the plasma membrane using HA- and c-Myc- tags, cells were blocked in 3% BSA and 5% normal donkey serum in 1X PBS without Triton X-100. Cells were incubated in primary antibodies at 4°C overnight, and washed with PBS three times before incubation in secondary antibodies at room temperature for 90 min, followed by 5 min DAPI staining with PBS washing. Slides were mounted with ProLong Gold mounting medium (Invitrogen) and images were taken using a confocal microscope (Carl Zeiss, LSM510).

## Results

### Analyses of jervell and lange-nielsen syndrome mutations

**Figure 1:**
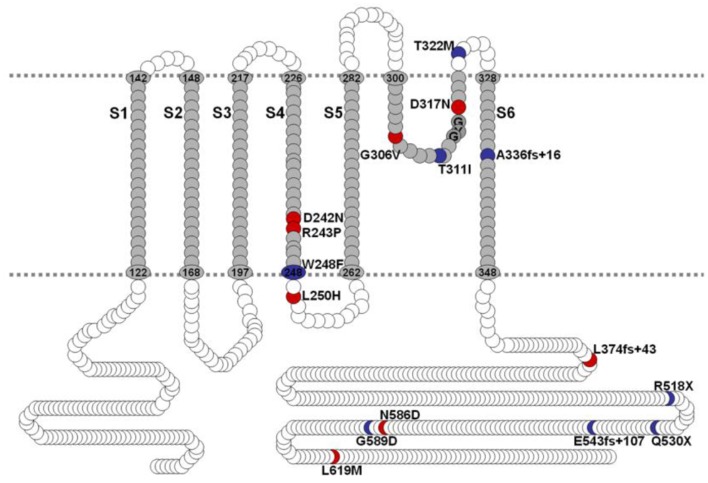
**Schematic diagram of hKv7.1**. JLNS mutations are indicated in blue, RWS, mutations are indicated in red. Amino acid numbers in transmembrane domains and P-loop regions were labeled based on UniProtKB/Swiss-Prot P51787. S4-S5 linker, channel gating; P-loop, permeation; C-terminal, regulation and tetramerization.

**Figure 2:**
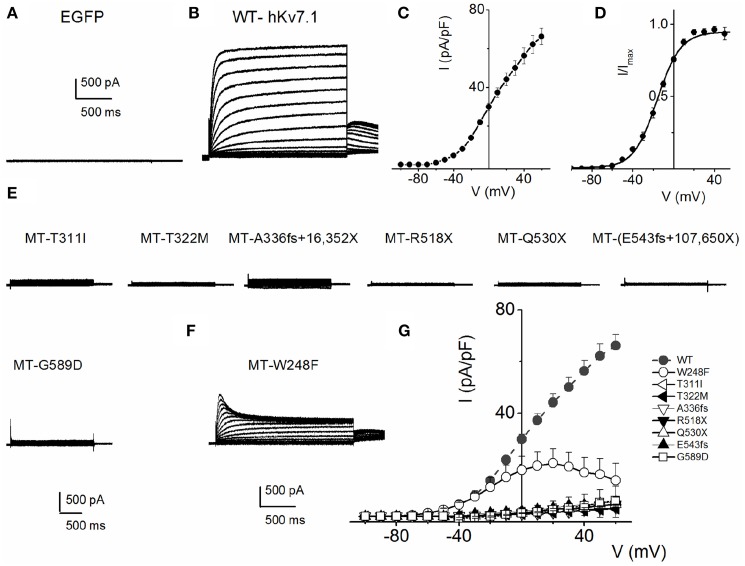
**Variations in current phenotypes of JLNS mutation in the hKv7.1 channel expressed in CHO cells A**. EGFP-alone transfected cells did not yield measurable current. Membrane K^+^ currents traces recorded from CHO cells after 24 h of transfection with EGFP, at a holding potential of −80 mV and step voltages ranging from −110 to 50 mV, with Δ V = 10 mV. **(B)** In contrast, robust outward K^+^ current traces ensued after wild-type (WT) hKv7.1 was transfected after 24 h. The current traces were generated using the voltage protocol described in **(A)**. Tail currents were recorded at −40 mV. **(C)** The corresponding current density-voltage relation shown was generated from *n* = 11 cells. The activation voltage of the expressed hKv7.1 current was ~-50 mV. **(D)** Voltage-dependence of steady-state activation generated from tail currents (I) as a ratio of the maximum tail current (I_max_). Each symbol represents mean current ratio obtained from 11 cells. Continuous line represents a single Boltzmann function fit to the data points. The half-activation voltage (V_1/2_) was −16.2 ± 0.9 mV, and the slope was e-fold for 11.4 ± 0.8 mV (*n* = 11). **(E)** Using similar activation voltage steps as described in **(A)**, CHO cells transfected with hKv7.1 channel mutants (MT), linked to JLNS, namely T311I, T322M, A336fs+16X, R518X, Q530X, E543fs+107X, and G589D, were evaluated. The MTs and resulting current traces are indicated. None of the seven mutants yielded detectable current, even after 48 h post-transfection. **(F)** Another recognized JLNS mutation is W248F. Unlike the other seven mutants, W248F yielded reduced current with pronounced inward rectification at positive voltage steps. Shown is a family of current traces recorded from transfected CHO cells (24-h-old). **(G)** Current density (in pA/pF)-voltage relations of the WT (in gray and dashed lines) and MT channels. Each symbol represents the mean of 11 cells.

Mutations of hKv7.1 that result in JLNS and RWS have been identified in families from America, Europe and Asia, transcending global ethnicities. Shown in Figure 1 is schematic representation of the topology of the hKv7.1 channel and the locations of recognized mutations, at the transmembrane segment 4 (S4), S4–S5 linker, the pore loop (S5–S6), S6 and the C-terminal that produce JLNS and RWS phenotypes. JLNS and RWS associated mutations have been color coded in blue and red, respectively. Table 1 outlines the global diversity of the ethnic background of patients with the type of disease, the domain of the protein and the associated exon for each mutation. To understand the mechanisms of the disease, we first determined the properties of the wild-type (WT) hKv7.1 channel by expressing it in CHO cells. Unlike EGFP-alone-transfected cells which did not yield measurable current (Figure 2A), WT hKv7.1 transfected cells held at a holding potential of −80 mV and stepped to voltages ranging from −100 to 60 mV produced outward K^+^ currents with mean maximum current density of ~70 pA/pF (Figure 2B). Analyses of the current density-voltage relationship and the steady state activation derived from the tail currents at −40 mV are shown in Figures 2C,D. The WT hKv7.1 channel currents have an activation voltage of ~−50 mV, half-voltage (V_1/2_) activation of −16.2 ± 0.9 mV, and slope factor (k) of 11.4 ± 0.8 mV (*n* = 11), using a single Boltzmann function to describe the activation curves. We examined the current phenotype of eight recognized mutations in hKv7.1 ascribed to JLNS using CHO cell expression strategy (Figures 2E–G). Seven (T311I, T322M, A336fs+16X, R518X, Q530X, E543fs+107X, and G589D) out of eight mutant channels yielded no visible K^+^ currents when expressed singly, even 48 h after transfection using voltage-clamp protocols, which is routinely employed to evoke currents in WT-hKv7.1 transfected cells (Figure 2B). Moreover, W248F mutant channel alone yielded reduced but robust outward K^+^ current compared to the WT and the other seven mutant channels, respectively. Additionally, W248F current profile showed inactivation at step voltages positive to 0 mV as depicted in Figure 2F and in the summary data of the current density-voltage relationship (Figure 2G).

**Figure 3:**
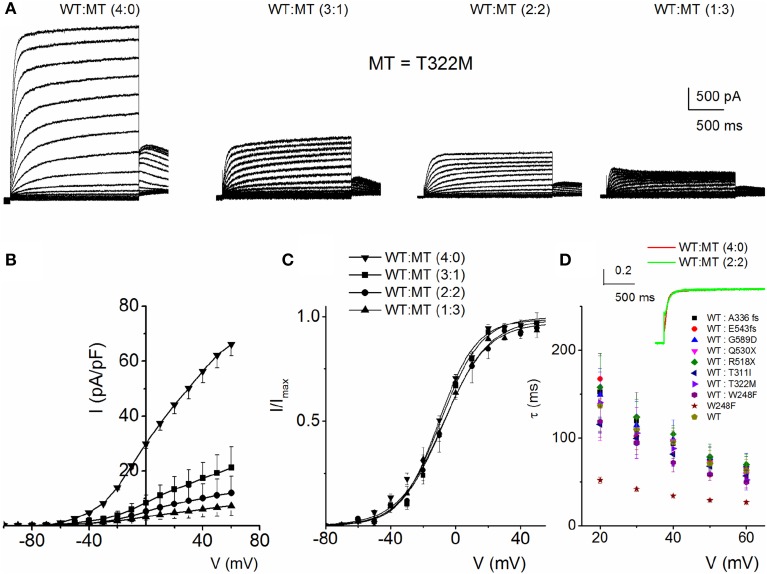
**Properties of heteromeric hKv7.1 and JLNS mutant channel currents. (A)** Macroscopic outward currents were recorded after expressing homomeric wildtype (WT:MT (4:0) channel, left panel) and upon co-expressing heteromeric hKv7.1 (WT) and MT T322M at different ratios (WT:MT) of 3:1 (left middle panel), 2:2 (right middle panel), and 1:3 (right panel). Representative current traces for a family of K^+^ currents obtained from a holding potential of −80 mV and stepped from −100 to 50 mV using Δ V = 10 mV are shown. The tail currents were elicited at −40 mV. **(B)** Plots of current density-voltage relation of currents derived from WT hKv7.1 alone (▾) and a combination of WT Kv7.1 and MT T322M (3:1, ◾ 2:2, ⚫, and 1:3, ▴). At a step potential of 0 mV, the current density (pA/pF) for WT hKv7.1 was 29.9 ± 1.9, as compared with the combined WT:MT (3:1) was 8.4 ± 2.8, WT:MT T322M (2:2) was 5.2 + 3.1 and WT: T322M (1:3) was 3.3 ± 1.3 (*n* = 11). **(C)** Co-expression of the MT T322M with WT-hKv7.1 channels did not alter the gating properties. Steady-state activation curves of WT Kv7.1 homomeric (▾) and heteromeric (3:1, ◾ 2:2, ⚫ and 1:3, ▴) currents are shown. Tail currents were measured immediately after pulsing to −40 mV, normalized to the largest tail current recorded, and plotted against the preceding pre-pulse voltages. Neither the mid-point (V_1/2_, in mV) nor the slope factors (k, in mV) were statistically different among the WT and cocktails of MT at different ratios. The V_1/2_ (in mV) and k (in mV) for WT Kv7.1 homomeric channel, and heteromeric WT:T322M (3:1; 2:2; 1:3) were: WT = −15.9 ± 1.1, 11.5 ± 0.5; WT:MT 3:1 = −16.0 ± 0.8, 11.7 ± 0.8; WT:MT 2:2 = 2:2 = −14.8 ± 1.8, 10.8 + 1.1; WT:MT 1:3 = 15.3 ± 0.6, 11.5 ± 0.8 (*n* = 11; *p* = 0.3, 0.4, 0.4, 0.5), respectively. respectively. **(D)** The currents derived from the WT hKv7.1 homomeric (green trace) and heteromeric channels (WT:T322M, 2:2; red trace) were normalized and superimposed, as shown in the inset. The time constants (τ) of mutations (MT, T311I, T322M, A336fs+16X, R518X, Q530X, E543fs+107X, and G589D) expressed jointly with the WT at a ratio of 2:2 were comparable, ranging from ~65 to 170-ms except W248F, which ranged from 27 to 52-ms, depending on the step voltage. Data were assembled from *n* = 11 Data were assembled from *n* = 11 cells.

To understand the cellular mechanism for JLNS, we co-expressed the MT and WT subunits. We were able to examine whether co-expression of the WT-hKv7.1 with the MT subunits was impactful. Figure 3 shows characteristic effects of the WT-hKv7.1:MT co-expression at different ratios. Since functional K^+^ channels form tetrameric complexes, we used different WT:MT ratios at 4:0, 3:1, 2:2, 1:3, 0:4. Current traces derived from WT:T322M at 3:1, 2:2 and 1:3 are shown (Figure 3A), and the corresponding current-density voltage relationship is plotted in Figure 3B. The summary data of the current density from the WT channel is included for comparison. We generated the steady-state activation curves from tail currents at −40 mV from the currents resulting from combinatorial expression of the WT and T322M. The data points were fitted with a single-state Boltzmann function (Figure 3C). Co-expression of the MT T322M with WT-hKv7.1 channels did not alter the gating properties (Figure 3C). For example, the V_1/2_ (in mV) and the slope factor k (in mV) for currents at these ratios (WT:T322M: 3:1, 2:2 and 1:3) were: WT:MT 3:1= −16.0 ± 0.8, 11.7 ± 0.8; WT:MT 2:2 = −14.8 ± 1.8, 10.8 ± 1.1; WT:MT 1:3 = −15.3 ± 0.6, 11.5 ± 0.8 (*n =* 11; *p* = 0.4, 0.4, 0.5). We plotted the time constant of activation (τ_act_) with respect to voltage for currents, resulting from combined expression of the individual eight JLNS mutants and WT subunits at the ratio of 2:2. Figure 3D shows that with the exception of W248F mutant, the τ_act_ of the WT currents were similar. The inset illustrates normalized current traces of WT current and WT-T322M-hybrid (2:2) current. The time constants (τ) of activation for all JLNS mutations in *KCNQ1* (MT, T311I, T322M, A336fs+16X, R518X, Q530X, E543fs+107X, and G589D) expressed together with the WT constructs at a ratio of 2:2 were comparable, ranging from ~65 to 170-ms except for W248F, which ranged from 27 to 52-ms, depending on the step voltage. Data were assembled from n = 11 cells (Figure 3D). These findings may reflect the possibility that the reduced current measured in CHO cell transfected with the WT and MT subunits was solely derived from assembly of homomeric WT channel currents. Previous studies showed that some *KCNQ1* JLNS mutations affecting the C-terminus of Kv7.1 channel, such as G589D, produced no current when expressed alone, but yielded measurable current when co-expressed with the WT subunit (Wang et al., 2011; Aromolaran et al., 2014).

**Figure 4:**
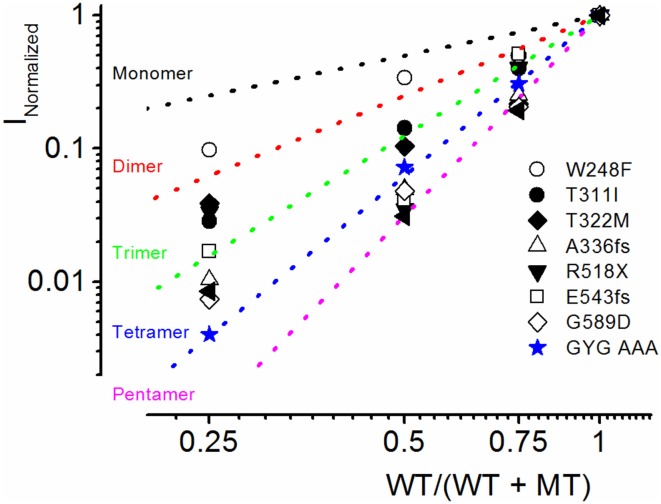
**Summary of experimental and predicted mechanisms of JLNS mutations**. The four α-subunits of K^+^ channels assemble to form a functional channel. The dotted lines denote the expected current reduction if the channels form monomers (black), dimers (red), trimers (green), tetramers (blue), and pentamers (magenta). We generated a dominant negative version of hKv7.1 in which the pore amino acids GYG (WT) were mutated to AAA (MT). The experimental data (in blue, star symbol) are in accord with the predicted relationship of tetramers, demonstrating that the WT_GYG_ and MT_AAA_ subunits co-assemble equally well with each other to form tetrameric functional channels. Current reduction of WT hKv7.1 by MT channels, T311I (⚫), W248F (○), T322M (♦), A336fs (▵) R518X (▾), E543fs (◻), G589D (◊) vs. WT/(WT + MT) ratio of DNA transfected. Reduction of WT hKv7.1 current was enhanced with decreasing WT:MT ratio. Whereas co-expression of WT hKv7.1 and the pore mutant channel AAA (dominant negative mutant) at different ratios yielded current magnitudes which confirmed the tetrameric structure of functional K^+^ channels, the experimental data from JLNS mutants deviated from the predicted relationship of tetrameric K^+^ channel.

Because combined expression of the WT and MT subunits yielded currents of sizable magnitude, by definition, these seven MTs cannot be classified as DN mutants. Nonetheless, assuming the predicted tetrameric structure of K^+^ channels (MacKinnon, 1991), we calculated the expected current magnitude when the WT channel is co-expressed with mutant subunits at different ratios. The data was compared to the expected results, when the mutant is operating as DN (mutations of the signature GYG_314−316_ to AAA_314−316_ in K^+^ channel pore) (MacKinnon, 1991; Xue et al., 2002). The expected results are shown with a dotted line in Figure 4. Consistent with our initial assertion, the experimental data points (in symbols) for the JLNS mutation suggested that none of the MT channels operated in a DN manner.

**Figure 5:**
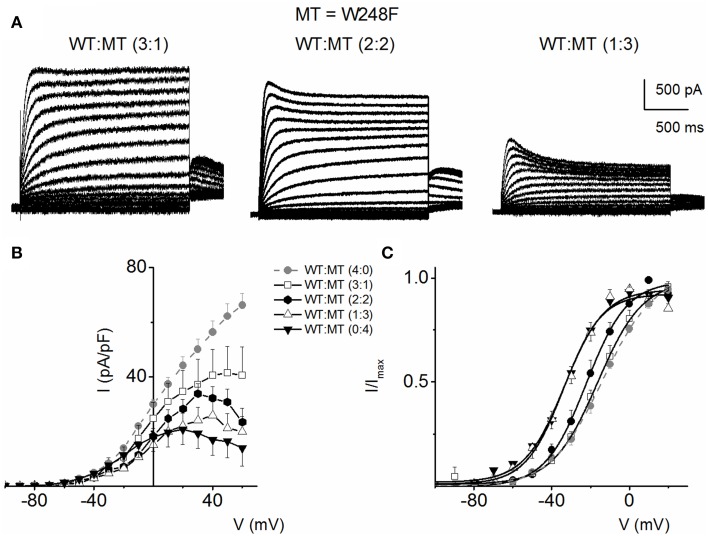
**Co-expression of JLNS mutant W248F and wild-type (WT) hKv7.1 channels. (A)** An example of outward current traces recorded from a holding potential of −80 to 50 mV using a voltage increment of 10 mV in depolarizing and hyperpolarizing step voltages. Current traces recorded CHO cells, which were transfected with WT-hKv7.1:MT-W248F (3:1, left panel), (2:2, middle panel), and (1:3, right panel). **(B)** Group data of current density-voltage curves (mean ± SD) for co-expression of WT-hKv7.1 alone (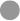) with WT-hKv7.1:MT-W248F (3:1, ◻), (2:2, ⚫), (1:3, ▵) and (0:4, ▾). In contrast to the WT channel-alone outward currents, co-expression of the WT and MT, as well as the MT channel by itself, yielded currents which showed robust inward rectification at depolarizing voltages greater than 0 mV step (data were generated from 14 cells for each group (3:1), (2:2), (1:3) and (0:4). **(C)** Summary data of the steady-state voltage-dependent activation of WT-hKv7.1 alone (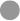), WT-hKv7.1:MT-W248F (3:1, ◻), (2:2, ⚫), (1:3, ▵) and (0:4, ▾). The V_*1*__/2_ of the steady-state activation curves of the five combinatorial expressions and the resulting currents were (in mV): WT-hKv7.1 alone (1:0), (3:1), (2:2), (1:3) and (0:4) −16.2 ± 0.9, −16.1 ± 0.8, −21.9 ± 1.6, −32.0 ± 0.7, −31.9 ± 1.2 (*n* = 9), respectively. The slope factors (k) of the resulting Boltzmann function curves were also not statistically different. The *k* values (in mV) for WT-hKv7.1 alone (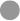),WT-hKv7.1:MT-W248F (3:1, ◻), (2:2, ⚫), (1:3, ▵) and (0:4, ▴) were 11.4 ± 0.8, 12.1 ± 0.5, 11.6 ± 1.1, 12.1 ± 0.7 and 12.2 ± 0.6 (*n* = 11; *p* = 0.7), respectively.

Figure 5 outlines our analysis of the resulting currents from combined expression of W248F and the WT channels. Exemplary current traces at different expression ratios are shown in Figure 5A. The corresponding current-density and voltage relationship is depicted in Figure 5B. The pronounced inactivation at positive voltages rendered the channel virtually non-functional. Comparing the WT (1:0) and WT:MT (3:1, 2:2, 1:3) currents, the steady-state voltage-dependent activation fitted with a Boltzmann function (V_1/2_) was shifted leftward, thus mutant channels were activated by more negative voltages compared to WT (WT-hKv7.1 alone (1:0), (3:1), (2:2), (1:3) and (0:1) −16.2 ± 0.9, −16.1 ± 0.8, −21.9 ± 1.6, −32.0 ± 0.7, −31.9 ± 1.2 (*n* = 9), respectively (Figure 5C).

### Functional properties of romano–ward syndrome mutations in hKv7.1

**Figure 6:**
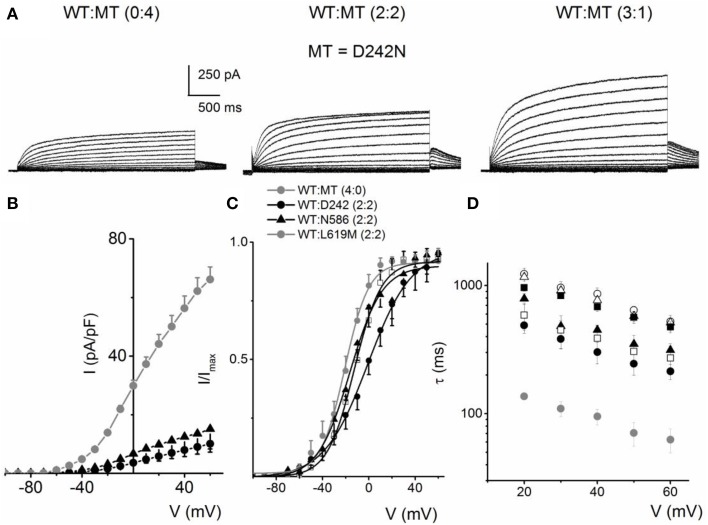
**Activation properties of homomeric and heteromeric hKv7.1 channel and RWS mutants, D242N, N586D and L619M currents**. **(A)** Whole-cell outward currents were recorded upon expressing hKv7.1 RWS mutant (MT-D242N) channel singly (left panel). Current traces recorded after co-expressing WT hKv7.1 and MT D242N at a ratio of 1:1 (middle panel). Representative current traces for a family of K^+^ currents obtained from a holding potential of −80 mV and stepped from −100 to 60 mV using Δ V = 10 mV are shown. The tail currents were elicited at −40 mV. Current traces recorded from CHO cells transfected with WT:MT-D242N ratio of 3:1 (right panel). **(B)** Plots of current density-voltage relation of currents derived from WT hKv7.1 alone (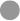) and a combination (ratio 2:2) of WT Kv7.1 and mutants (MT-D242N, ⚫, MT-N586D, ▴, and MT-L619M, ◻). **(C)** Heteromeric association between the WT hKv7.1 and MT-D242N, N586D and L619M at a ratio (2:2) altered the voltage-dependent activation of the ensuing currents. Steady-state activation curves of hKv7.1 alone (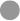) and after suppression by co-joint expression with the MT channels are shown. Tail currents were measured immediately after pulsing to −40 mV, normalized to the largest tail recorded, and plotted against the preceding pre-pulse voltages. The midpoint (V_1/2_, in mV) and the slope factors (k, in mV) were as follows: V_1/2_ and k for WT-hKv7.1 alone and in combination WT-hKv7.1:D242N, WT-hKv7.1:N586D, and WT-hKv7.1:L619M were −16.3 ± 0.8, 11.4 ± 0.9; −1.0 ± 1.5, 13.1 ± 1.1; −12.8 ± 1.2, 11.7 ± 0.6; −13.2 ± 0.9, 11.8 ± 0.9 mV (*n* = 10), respectively. **(D)** Homomeric MT channels and combined expression of the MT channels (D242N, N586D, and L619M) and WT hKv7.1 produced ~3-8-fold increase in the time constant of activation (WT-hKv7.1 (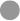), D242N (○), N586D (▵), L619M (◾), WT-hKv7.1:D242N (2:2) (⚫), WT-hKv7.1:N586D (▴), WT-hKv7.1:L619M (◻) (*n* = 11 cell for each group).

We evaluated eight mutants that have been reported to be associated with RWS. These mutations were identified by their location at; the S4 and S4–S5 linker (D242N, R243C, L250H), the pore loop (G306V, D317N) and the C-terminal (L374fs+43X, N586D, L619M) of the hKv7.1 channel, similar to the identified sites for JLNS mutations. Each mutant was generated and expressed singly, as well as co-jointly, with the WT subunit in tetrameric ratios as described for JLNS mutations. Figure 6 shows an example of RWS mutant, D242N that has a reduced current compared to the WT channel. The current traces and the respective ratios are indicated (Figure 6A). As the ratio of the WT:MT subunit increased, the magnitude of the whole-cell K^+^ current was enhanced. However, the magnitude of the current remained stunted compared to the WT channel currents. The RWS mutants, D242N, N586D, and L619M, exhibited similar features. The current-density voltage relationship of the three RWS mutant currents is summarized in Figure 6B, which demonstrates N586D had slightly elevated current compared to D242N and L619M. Comparing the WT and MT currents, the steady-state voltage-dependent activation fitted with a Boltzmann function were moved rightward as follows: V_1/2_ (in mV) and k (in mV) for WT-hKv7.1 alone and in combinations of WT-hKv7.1:D242N, WT-hKv7.1:N586D, and WT-hKv7.1:L619M (all in 2:2 ratios) were; −16.3 ± 0.8, 11.4 ± 0.9; −1.0 ± 1.5, 13.1 ± 1.1; −12.8 ± 1.2, 11.7 ± 0.6; −13.2 ± 0.9, 11.8 ± 0.9 mV (*n* = 10), respectively (Figure 6C). An additional finding in the three mutants is that the τ s of activation were substantially slower than the WT current (Figure 6D). In contrast to our observation, Aromolaran et al. (2014) reported that L619M did not produce any current when expressed alone in the CHO cells, but a significant amount of current was observed when this mutant was co-expressed with WT and KCNE1.

**Figure 7:**
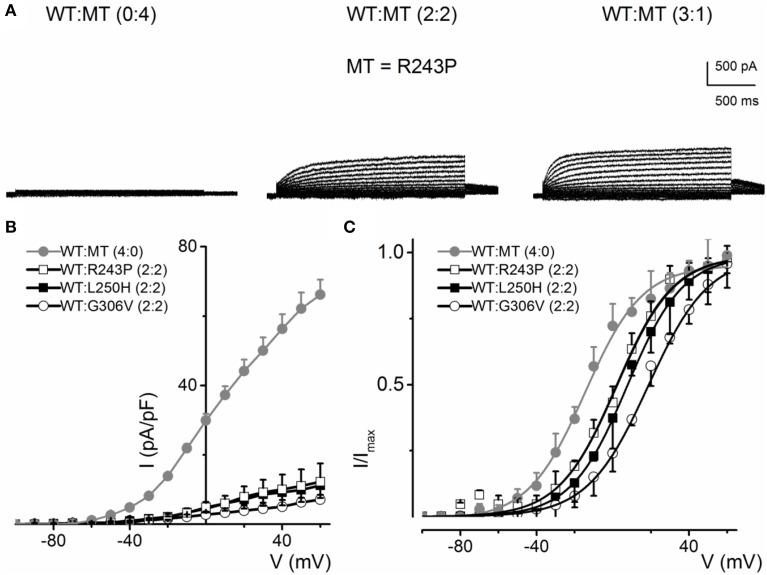
**Current phenotype of homomeric and heteromeric hKv7.1 channel and RWS mutants, R243P, L250H and G306V channels**. **(A)** CHO cells were transfected with RWS mutant R243P DNA. Whole-cell outward currents were recorded after 48 h. Cells were held at −80 mV and stepped to voltages ranging from −100 to 60 mV using Δ V = 10 mV. Homomeric MT channel R243P did not yield outward currents (left panel). Similar currents were obtained for homomeric L250H and G306V MTs. However, co-expression WT hKv7.1 and MT R243P at a ratio of 2:2 (middle panel) yielded reduced current magnitude (middle panel), which was enhanced further after increasing the ratio of the WT-hKv7.1:R243P (3:1, right panel). **(B)** Plots of representative current density-voltage relation of currents derived from WT hKv7.1 alone (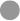) and a combination (ratio 2:2) of WT Kv7.1 and MTs (MT-R243P ◻, MT-L250H ◾, and MT-G306V ○). **(C)** Co-expression of WT hKv7.1 and MT-R243P, L250H and G306V at a ratio (2:2) produced a right-ward shift in the voltage-dependent activation of the resulting currents. Steady-state activation curves of hKv7.1 alone (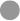) and after suppression by co-expression with the MT channels are shown. Tail currents were measured at −40 mV, and normalized to the largest tail current magnitude, and plotted against the preceding pre-pulse voltages. The midpoint (V_1/2_, in mV) and the slope factors (k, in mV) were as follows: V_1/2_ and k for WT-hKv7.1 alone and in combination WT-hKv7.1:R243P, WT-hKv7.1:L250H, and WT-hKv7.1:G306V were −15.8 ± 1.2, 11.8 ± 0.9; 2.5 ± 1.1, 12.4 ± 0.6; 7.6 ± 2.2, 12.7 ± 1.0; 15.7 ± 2.4, 12.8 ± 1.6 mV (*n* = 11), respectively.

Some KCNQ1 RWS mutants (R243P, L250H and G306V) were non-functional when transfected alone, but showed stunted currents when co-transfected with WT. The family of current traces in Figure 7A is an example of the phenotypic outcome of expression of R243P alone and at different ratios with the WT channel. The current density-voltage relationships of the three mutants suggest that although the mutant alone may be non-functional, the presence of the WT channel may render or facilitate mutant subunit functional (Figure 7B). The steady-state voltage-dependent activation of current generated from conditions when the WT:MT ratio is (2:2) were variable (Figure 7C). The midpoint (V_1/2_, in mV) and the slope factors (k, in mV) were as follows: V_1/2_ and k for WT-hKv7.1 alone and in combination (2:2) WT-hKv7.1:R243P, WT-Kv7.1:L250H, and WT-hKv7.1:G306V were −15.8 ± 1.2, 11.8 ± 0.9; 2.5 ± 1.1, 12.4 ± 0.6; 7.6 ± 2.2, 12.7 ± 1.0; 15.7 ± 2.4, 12.8 ± 1.6 mV (*n* = 11), respectively. The steady-state voltage-dependent activation was shifted rightward. Therefore, mutant channels were activated in more positive voltages in compare to WT (Figure 7C). Our results is in agreement with study conducted by Chouabe et al. (2000) where they showed that RWS mutants such as R243H and R533W not only reduced the amount of current, but also shifted the voltage-dependent activation of the channel to the more positive voltages.

**Figure 8:**
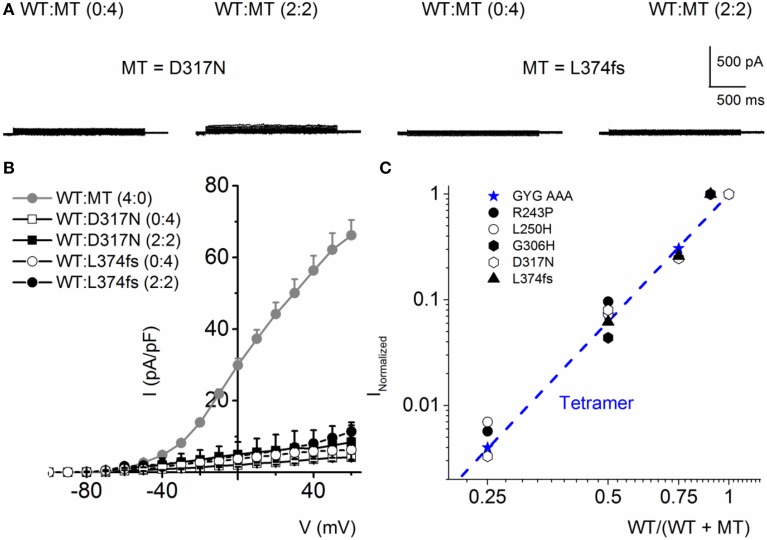
**RWS MT D317N and L374fs+43X exhibit strong dominant negative effect on WT hKv7.1 channel functions. (A)** Characteristic current traces recorded from a holding potential of −80 mV and activated from −100 to 40 mV with Δ V of 10 mV. The MT subunits (D317N and L374fs+43X) when expressed alone did not produce measurable currents. Co-expression of the WT-hKv7.1 and MT subunits yielded little or no measurable currents. **(B)** Current density (in pA/pF)-voltage relations of the WT and MT channels and after co-expression of both subunits as indicated. Each symbol represents the mean of 11 cells. **(C)** Using similar analyses as described in Figure 4, we show that the RWS MTs indicated suppressed the WT-hKv7.1 current in a DN fashion. We used the peak current magnitude at 40 mV step voltage to perform the analyses.

The mutants, D317N and L374fs+43X, were non-functional when expressed alone and even in the presence of the WT subunit at variable ratios, the resultant current remained substantially reduced (Figures 8A,B). These finding is in agreement with previous finding showing the dominant-negative effect of some RWS mutants on WT subunits (Chouabe et al., 2000; Thomas et al., 2005; Spatjens et al., 2014).

**Figure 9:**
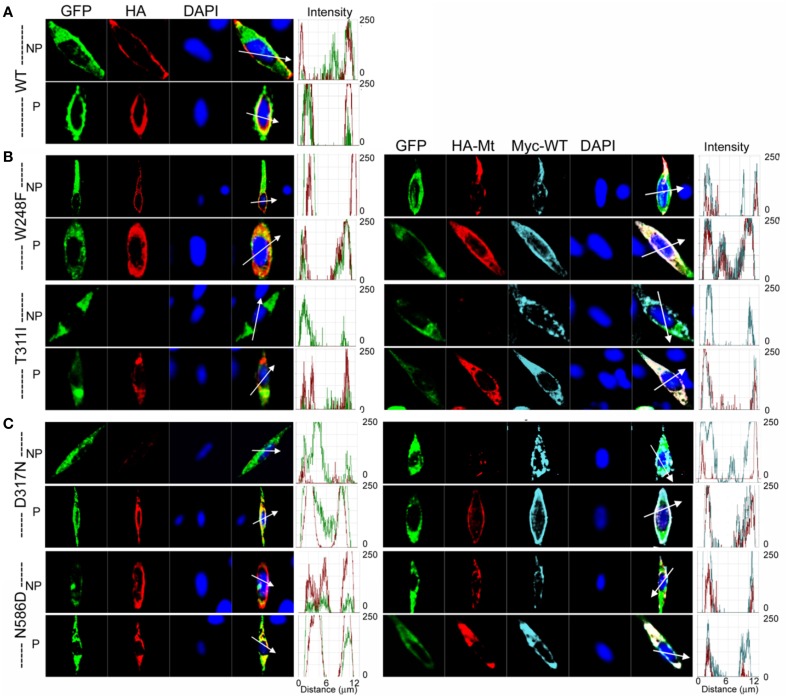
**Detection of cell surface expression using epitope tagged hKv7.11**. HA- or c-Myc-epitope tag was inserted with an extension of ClC-5 chloride channel D1–D2 loop sequences into the extracellular loop between S1 and S2-transmembrane domains. Farnesylated GFP was used as a plasma membrane binding protein. **(A)** Wild-type (WT); Anti-HA antibody stained wild-type hKv7.1 protein expressed on the cell surface in non-permeabilized condition (NP), and stained both the cell surface and the cytoplasm expressed proteins in permeabilized condition (P). **(B)** JLNS mutants (MT); One MT out of 8, W248F, was detected on the cell surface, while T311I was not detected on the cell surface (left panel). When the MT subunit was co-transfected with WT subunit, T311I MT failed to localize on the plasma membrane while WT subunits were detected in NP conditions. Permeabilized cells expressing both WT and MT subunits (right panel). **(C)** RWS MTs; All of the RWS MTs were detected on the cell surface with different levels of intensities. *Left panels*: Fluorescent intensities of GFP (*green*) and hKv7.1 channel (*red*) were plotted against the distance, which was marked in the merged image with the *white arrow*. DAPI (*blue*; 4′, 6-diamidino-2-phenylino-2-phenylindole) is a fluorescent stain that binds strongly to A-T rich regions in DNA, and was used as nuclear stain. *Right panels*: HA-MT subunit (*red*) and c-Myc-WT subunit (*cyan*) were plotted against the distance.

Further analysis using methods described in Figure 4 was consistent with the revelation that D317N and L374fs were indeed operating strongly as bona fide DN mutants (Figure 8C). The analysis described in Figures 4, 8 regarding the predicted current magnitude after co-expression of MT-hKv7.1 and the WT subunits pre-supposes that the synthesis and membrane translocation of the MT and WT subunits occur independently. Further, assembly of the subunits is stochastic (MacKinnon, 1991; Xu et al., 2007). To understand the cellular mechanisms that explain why the MT-hKv7.1 channel expressed in CHO cells did not yield current but may appear to be functional in the presence the WT subunit, we decorated the MT, WT and subcellular organelles with distinct labels. Cell-surface expression of WT- and MT-hKv7.1 channel subunits were assured with HA- and c-Myc-epitope tags in the extracellular loop between S1 and S2 transmembrane domains, and farnesylated GFP was used as the reporter gene, which is plasma membrane-bound. The HA- and c-Myc-tagged WT hKv7.1 channels were labeled in the plasma membrane in non-permeabilized (NP) cells (Figure 9A) as compared with hKv7.1 antibody labeling, which was directed against the intracellular C-terminal of the channel, which did not show positive reactivity in NP condition (data not shown).

**Figure 10:**
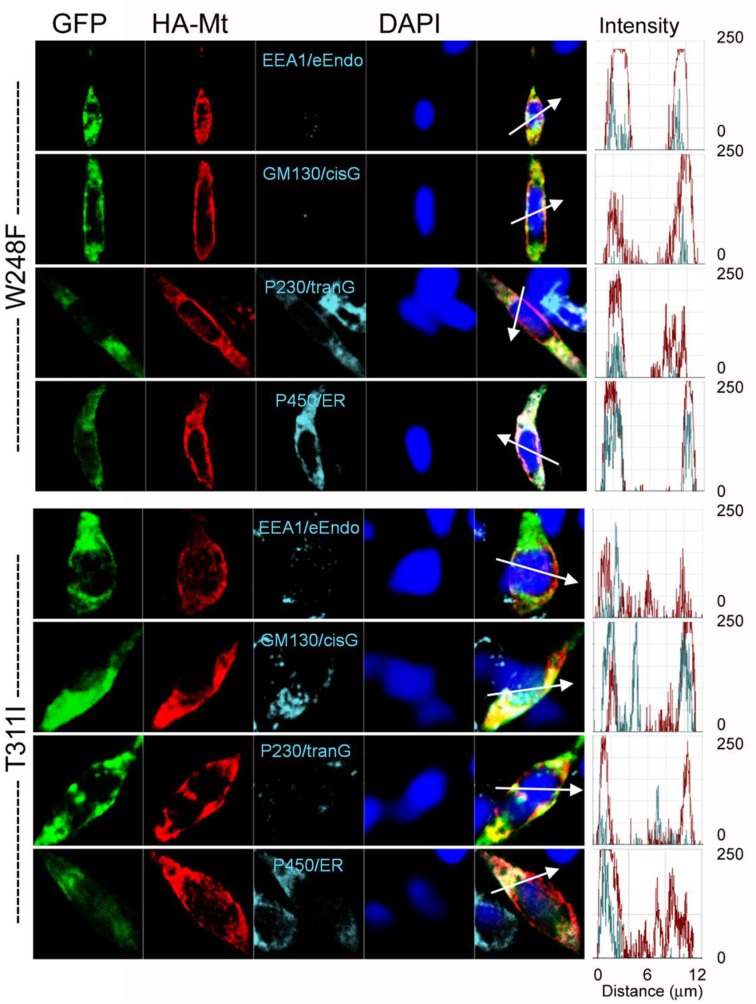
**Detection of subcellular localization of mutant hKv7.1 channels**. HA-tagged MT Kv7.1 channels and subcellular organelles were double-stained in permeabilized cells (*P450/ER*, anti-cytochrome P450 as endoplasmic reticulum marker; *P230/tranG*, anti-golgin A4 (P230) as trans-Golgi marker; *GM130/cisG*, anti-golgin A2 (Golgi matrix protein of 130 kDa) as cis-Golgi marker; *EEA1/e-Endo*, anti-early endosome antigen 1 as endosome marker). Fluorescent intensities of MT hKv7.1 (*red*) and subcellular organelles (*cyan*) were plotted against the distance, which was marked in a merged image with a *white arrow*. The overlap pattern of red and cyan signals showed different levels of co-localization between organelles.

The data from JLNS mutant subunits did not provide evidence for the mutants acting as DN-mutants. Instead, it suggested that the ensuing currents from co-expression of the WT:MT (2:2) subunits exhibited voltage-dependent and kinetic properties that mirrored the WT homomeric currents. Thus, we assessed the plasma membrane and cellular localization of the WT and MT subunits (Figures 9, 10). Here, we show typical example of JLNS mutant, T311I that did not yield functional currents, when expressed singly (Figure 9). The MT subunit was synthesized but was not localized in the plasma membrane, suggesting that the JLNS mutant channels had an impaired trafficking mechanism (Figure 9B). Sub-cellular identification of JLNS mutant, T311I, suggested that they were trapped in the ER and cis-Golgi (Figure 10). The supplementary Figures S1, S2 demonstrate localization of the other six JLNS mutants, T322M, A336fs+16X, R518X, Q530X, E543fs+107X, and G589D. These JLNS mutants, when expressed singly, showed minimum-to-absent plasma membrane localization (S1), indicating defective membrane trafficking. Similar to the data described for T311I, the other six JLNS mutants were localized mainly in the ER and cis-Golgi (S2). In keeping with the functional data, and as demonstrated in Figure 9, the WT and W248F channels were synthesized, trafficked, and localized in the plasma membrane. As it turns out, the RWS mutants had weak membrane expression (Figure 9C, Figure S3).

Wang et al. (2011) reported that the JLNS T2C mutant exhibited impaired cell-surface expression due to trafficking defects, resulting in non-functional channel. Our results showing impaired cell-surface expression due to a trafficking deficit in the G589D mutant is in agreement with a similar study conducted by Aromolaran et al. (2014).

## Discussion

In this study, we investigated the biophysical and cellular effects of seemingly similar mutations in *KCNQ1* which result in phenotypic differences between JLNS and RWS. We selected mutations which were identified in JLNS vs. RWS patients, but in most cases their cellular mechanisms were unknown. In order to provide mechanistic insight on the functional outputs of JLNS vs. RWS mutations, we chose RWS mutations adjacent or spatially close to JLNS mutation sites at the S4-S5-linker, the pore loop (P-loop) and the C-terminus because previous studies have identified these sites as being responsible for the voltage-dependent activation, permeation and modulation/tetramerization of K^+^ channels, respectively. The primary aim of this study was to determine whether the cellular mechanisms for the diverse phenotypic outcome of *KCNQ1* mutations are dependent on the tissue-specific function of the channel and/or specialized functions of the channel.

We report that: (1) with the exception of W248F, which is located at the end of the S4 domain, all JLNS mutants, pore loop (T311I and T322M) and C-terminal (A336fs+16X, R518X, Q530X, E543fs+107X, and G589D) yielded no current when expressed as homotetramers; (2) by contrast, homomeric W248F produced a reduced current magnitude but portrayed rapid inactivation at positive voltages, invalidating the channel as an effective outward K^+^ current; (3) using immunofluorescence strategies to label the channels and subcellular organelles, we observed that with the exception of W248F, JNLS mutants do not reach the plasma membrane but remain in the ER and cis-Golgi; (4) W248F is translocated into the membrane unimpeded; (5) moreover, the remaining JLNS mutant subunits, when co-expressed with WT subunit remain trapped in organelles in the cytoplasm. Meanwhile, the WT subunits were detected in the plasma membrane. Thus, when co-expressed WT: MT, the measured current is likely to originate from homomeric WT-subunit assembly, in keeping with the findings illustrated in Figure 3. (6) RWS mutations have varied phenotypic outcomes, consisting of those with reduced currents and altered voltage-dependent activation with and without a co-transfected WT subunit. RWS mutants impeded membrane trafficking, as well.

The JLNS mutation at the end of S4, W248F, did not show impaired trafficking and yielded reduced outward current with profound inward rectification at positive voltages. The S4 segment, the voltage sensor of K^+^ channels (Jiang et al., 2003; Gagnon and Bezanilla, 2010; Wang et al., 2013), undergoes asymmetric charge movement, which is represented as fast-on and slow-off gating currents (Perozo et al., 1993; Bezanilla et al., 1994; Stefani et al., 1994). Amino acid residues in the S4–S5 linker have been shown to stabilize the channel in the open state (Batulan et al., 2010), raising the possibility that W248F may imbed structural changes that will ultimately destabilize the open state of the channel, leading to the apparent inactivation of the channel. The pore and C-terminal mutants associated with JLNS to a rough approximation appear similar, showing clear impairment in channel trafficking. The precedence for K^+^ channel pore mutations and diminished channel trafficking is set in Kv7.4 mutation G285C, which results in an autosomal dominant form of progressive hearing loss (DFNA2). Previous studies have demonstrated that co-expression of the wild-type and the mutant subunits at different ratios produced current magnitudes which were not in keeping with the G285C serving as a DN mutant (Kubisch et al., 1999; Xu et al., 2007; Mencia et al., 2008). Indeed, replacement of glycine with cysteine at the pore region of hKv7.4 alters channel trafficking and plasma membrane expression (Kim et al., 2011). The C-terminal A-domain or coiled-coil domain (CCD) has been shown to drive tetrameric assembly, stability and selectivity of multimerization (Jenke et al., 2003; Howard et al., 2007). The subunit assembly domain resides at the C-terminal segment 589–620. The JLNS mutants R518X and Q530X are expected to have a truncated C-terminus missing the A-domain. G589D occurs at the onset of the assembly domain and it is predicted to disrupt channel multimerization. Finally, E543fs+107X have distinct amino acid sequence by frame shift that is not in keeping with the CCD. Thus, our findings that these mutations revealed robust mutant subunit trapping in the ER and Golgi, dovetail well with the conclusion that the mutant subunit trafficking defect is a major factor in the disease phenotype. The reduced current recorded after joint expression of the WT and mutant subunits likely originates from independent homomeric assembly of the WT subunits.

All RWS mutations examined yielded stunted currents when expressed jointly with the WT channel. Qualitatively, D242N, R243P, L250H, G306V, L374fs+43, 352X, N586D and L619M had weak expression levels compared to the WT channel. Consistent with the S4 segment and S4–S5 linker serving as the voltage sensor, charged to neutral amino acids substitution in D242N, R243P and vice versa in L250H, distal to the S4 segment have voltage-dependent properties that were significantly weaker than the WT channel. The current seen in pore mutants G306V and D317N is conceivable since they do not alter the selectivity filter (GYG) but are expected to produce slight changes in the pore structure. In contrast to the C-terminal mutants of the JLNS, which have either a truncated A-domain or an altered overall apparent structure of the assembly domain, the RWS mutants had conserved mutations in the A-domain (L619M) or point mutations (N586D) that occur proximal to the assembly domain. The findings suggest that the RWS mutants form multimers and are translocated into the membrane.

To ensure normal cardiac functions, the regular rhythmic profile of a variety of specialized chamber-specific cardiac cells has to be coordinated by time-dependent changes in ionic conductances (Kurokawa et al., 2001). Besides their role as determinants of the resting potential, currents derived from Kv7.1 channels and their associated β-subunit, KCNE1 (Chouabe et al., 2000), regulate the duration of the plateau phase of cardiac action potentials (Noble and Tsien, 1969; Sanguinetti, 1999). Because of time-dependent requirements of Kv7.1/KCNE1-mediated currents, mutations in the α- or β-subunits and the ensuing altered current magnitude delay repolarization and cause different forms of LQTS (LQT1-LQT5) (Sanguinetti, 1999).

In the inner ear, there is a unidirectional flux of K^+^ from Spiral Ligament to the Marginal Cells (MC). The K^+^ flux is maintained by Kv7.1 channels, coating the apical membrane of MC and the continuous K^+^ flux across the MC apical membrane results in the exceptionally high [K^+^] in the endolymph (~150 mM).

K^+^ cycling in the cochlear duct maintains the high throughput rate of K^+^ across the lateral wall, which confers the Endococlear Potential (EP). Thus, the essential requirement for the generation and maintenance of the EP is the continuous flow of K^+^ which is not time-dependent, in contrast with cardiac where the timing of activation and deactivation of I_KS_ is essential for cardiac function.

Our results demonstrate that JLNS mutations result in non-functional channels with impaired membrane trafficking, so the phenotypic consequences are observed in both inner ear and heart. In contrast, RWS mutations usually lead to functional channels but with significantly reduced current, therefore the phenotypic consequence of mutation is primarily observed in heart where timing of currents is most critical. Moreover, the K^+^ channel tetramerization was reported to be stochastic (Kubisch et al., 1999), therefore all the different combinations of WT and MT subunits are seen in RWS patients, which apparently is sufficient to maintain EP in the inner ear.

### Limitations of the study

The primary focus of this study was on the effects of specific mutations of the pore-forming α-subunit on the biophysical properties of hKv7.1. Relying on only the outcomes of the features of α-subunit, it can be inferred that the tissue-specific functions of the channel in the inner ear lateral wall and the heart may trump on nuisances of biophysical alterations in channel function in a specific class of disease. Moreover, almost invariably, the physiological and pathologic properties of Kv7 channels can be dictated by the β-subunit (e.g., KCNE subunits) and other auxiliary binding partners, limiting the scope of the present study. For example, previous reports have indicated that in the presence of KCNE1, W248F induced a rightward shift in the voltage-dependent activation of hKv7.1 currents (Franqueza et al., 1999). In contrast as shown in this study (Figure 5), without KCNE1, the voltage-dependent activation moved leftward. Previous study showed that Kv7.1 voltage sensors move independently, and the channel can conduct ions even before all the voltage sensors move. Some mutation cause the voltage sensors move in more depolarize voltage and as a result channel open in more negative voltage as seen in W248F mutant (Osteen et al., 2012).

Since diverse and multiple β-subunits can associated with the pore-forming subunit, compounded by the fact that different α-subunits can form functional heteromultimers in native cells, interpretation of results from heterologous expression systems can be made with these caveats in mind. Yet, studies in expression systems provide a powerful and robust way to understand the biophysical properties of a channel in a reduced structure. Analyses performed for data in Figures 4, 8, explicitly assume that MT and WT subunits have equal probability of assembly into a channel. However, if a mutant subunit impairs channel trafficking, such that the probability of WT subunit homomeric channel is conditional on the MT subunit then the analyses will fail to predict the expected outcome. Thus, while the present report provides a glimpse of the possible mechanisms for JLNS and RWS, further studies may be required to understand the details fully.

### Conflict of interest statement

The authors declare that the research was conducted in the absence of any commercial or financial relationships that could be construed as a potential conflict of interest.
