# Supplementary material for: Cellular mechanisms of mutations in Kv7.1: auditory functions in Jervell and Lange-Nielsen syndrome vs. Romano–Ward syndrome
Source: Front Cell Neurosci. 2015 Feb 6;9:32. doi: 10.3389/fncel.2015.00032 (PMC4319400; doi:10.3389/fncel.2015.00032)
Supplement: Supplementary file 1 [file Image1.PDF]

Supplementary figure S1

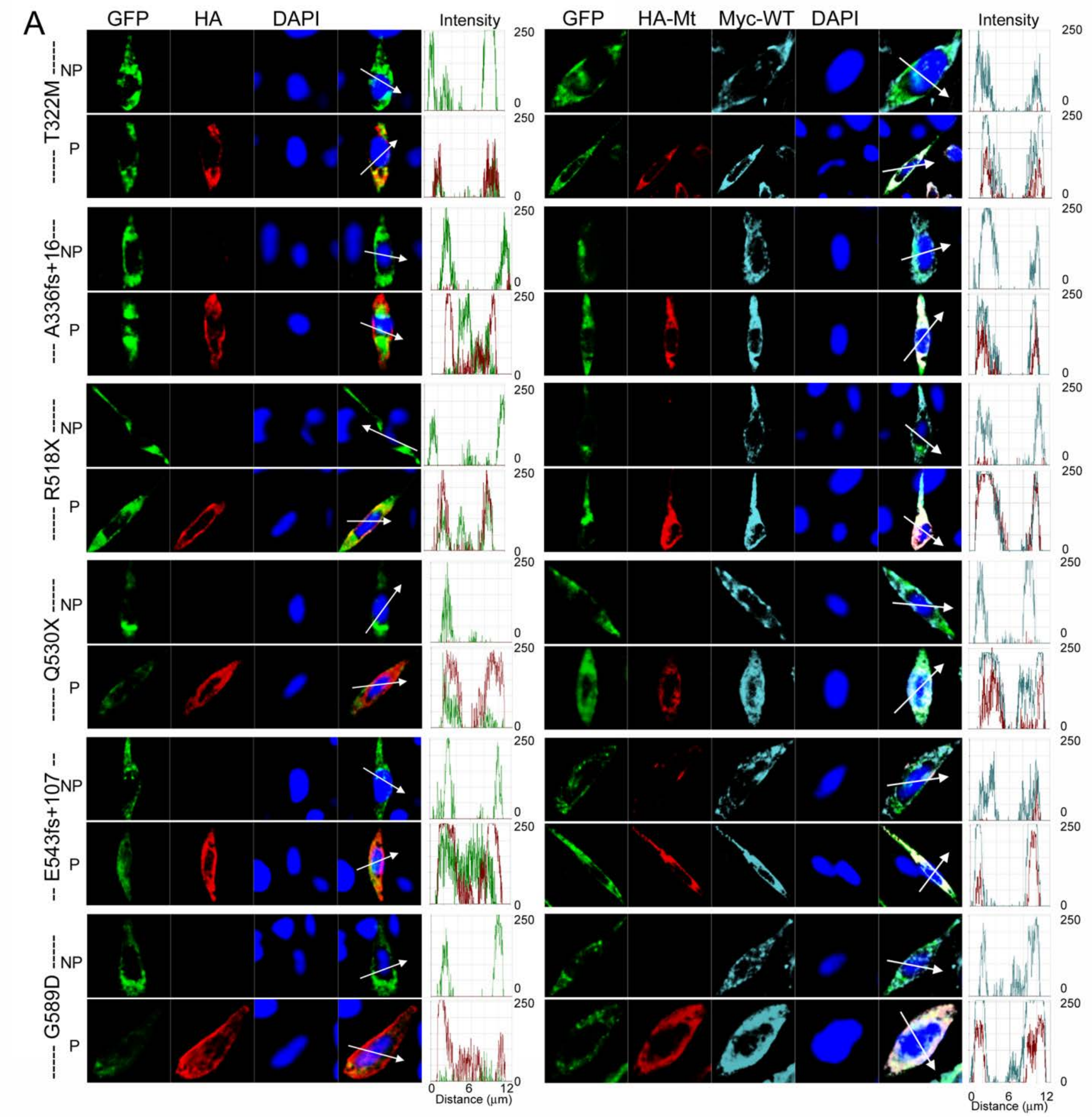

Detection of cell surface expression using epitope tagged hKv7.1

JLNS mutants (MT)s were not detected on the cell surface (left panel) even when the MT subunit were co-transfected with WT subunit.
