# Supplementary material for: Cellular mechanisms of mutations in Kv7.1: auditory functions in Jervell and Lange-Nielsen syndrome vs. Romano–Ward syndrome
Source: Front Cell Neurosci. 2015 Feb 6;9:32. doi: 10.3389/fncel.2015.00032 (PMC4319400; doi:10.3389/fncel.2015.00032)
Supplement: Supplementary file 2 [file Image2.PDF]

Supplementary figure S2

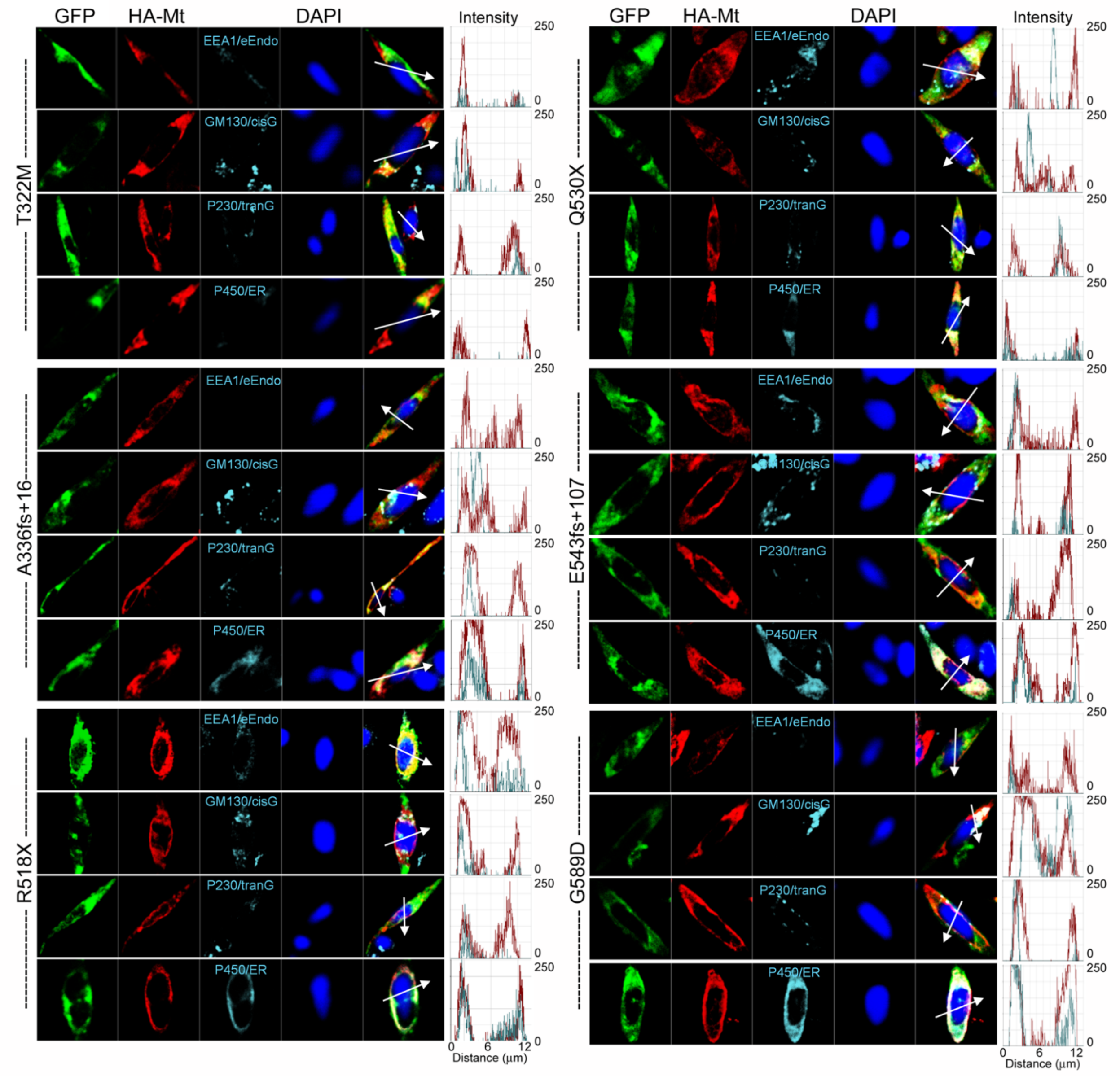

Detection of subcellular localization of mutant hKv7.1 channels

HA-tagged JLNS MT Kv7.1 channels and subcellular organelles were double-stained in permeabilized cells (p450/Er, anti-cytochrome P450 as endoplasmic reticulum marker; P230/tranG, anti-golgi A4 (p230) as trans-Golgi marker; GM130/cisG, anti-golgi A2 (Golgi matrix protein of 130 kDa) as Cis-Golgi marker; EEA1/e-Endo, anti-early endosome antigen 1 as endosome marker). Florescent intensities of MT hKv7.1 (red) and subcellular organelles (cyan) were plotted against the distance, which was marked in a merged image with a *white arrow*. The overlap pattern of red and cyan signal showed different levels of co-localization between organelles. Most of the MT channels were identified in ER and Cis-Golgi.
