# Supplementary material for: Cellular mechanisms of mutations in Kv7.1: auditory functions in Jervell and Lange-Nielsen syndrome vs. Romano–Ward syndrome
Source: Front Cell Neurosci. 2015 Feb 6;9:32. doi: 10.3389/fncel.2015.00032 (PMC4319400; doi:10.3389/fncel.2015.00032)
Supplement: Supplementary file 3 [file Image3.PDF]

Supplementary figure S3

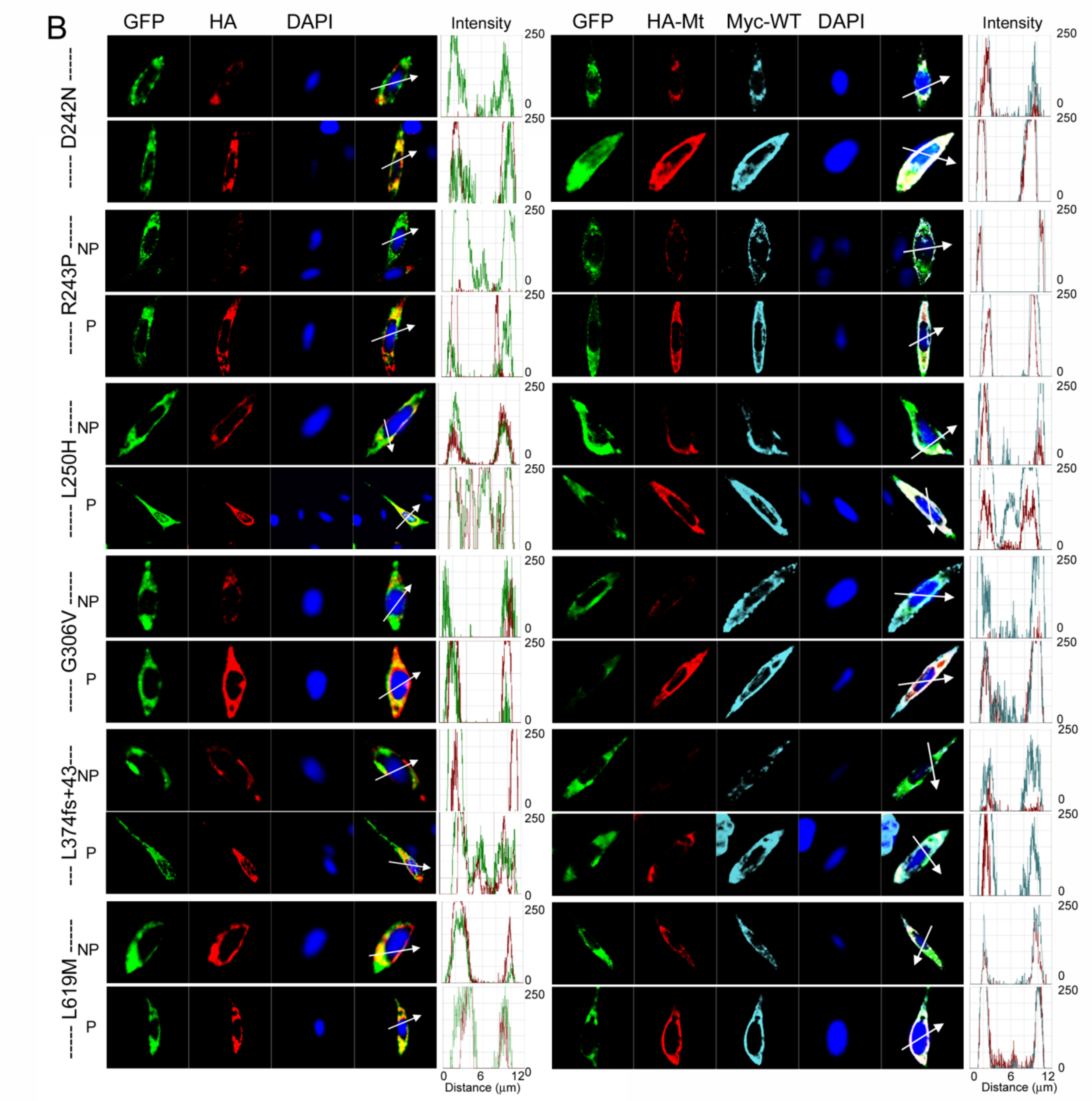

Detection of cell surface expression using epitope tagged hKv7.1

RWS mutants (MT)s detected on the cell surface (left panel), but they had weak membrane expression compare to WT subunit even when the MT subunit were co-transfected with WT subunit (right panel).
